# Chronic wasting disease as a model for human prion therapy

**DOI:** 10.1080/19336896.2025.2510665

**Published:** 2025-05-24

**Authors:** Michael Bordonaro

**Affiliations:** Department of Medical Education, Geisinger Commonwealth School of Medicine, Scranton, PA, USA

**Keywords:** Cervids, chronic wasting disease, deer, prion, therapy

## Abstract

Prion diseases, also known as transmissible spongiform encephalopathies (TSEs), are fatal neurodegenerative disorders that result from abnormally folded prion proteins. These disorders can be sporadic, acquired, or genetic. Acquired TSEs can be found in a number of animal species including sheep (scrapie), cows (bovine spongiform encephalopathy), and cervids (chronic wasting disease). Chronic wasting disease (CWD) is unusual in that it is found in free ranging animals in their natural environment. There has been heretofore no reported cases of CWD being transmitted to humans; however, the possibility of future adaption for human transmission exists. Several novel approaches for the prevention and treatment of prion disease in humans are under development, including knockdown of endogenous prion expression or overexpression of dominant negative prion forms. Here, I propose that CWD, as a naturally occurring prion disease, should be considered an important testing target for such therapies, which can demonstrate efficacy outside of controlled laboratory environments. Further, from the ethical standpoint of reducing animal suffering, decreasing the CWD burden in cervid populations is highly desirable. Finally, lowering CWD incidence can reduce future possibilities of transmission to humans or to other animal species.

## INTRODUCTION

Prion diseases (transmissible spongiform encephalopathies or TSEs) are invariably fatal neurodegenerative disorders [1,2] that result from the downstream consequences of the accumulation of abnormally folded prion proteins (PrPs). TSEs can be sporadic; genetic, with an age-related onset [1–3]; or acquired.

Besides iatrogenic prion disease and variant Creutzfeldt Jakob Disease (vCJD) in humans, acquired TSEs can be found in a number of animal species including sheep (scrapie), cows (bovine spongiform encephalopathy, transmitted to humans as vCJD), and cervids (chronic wasting disease). Having an international distribution and currently concentrated in North America [4–10], chronic wasting disease (CWD) manifests in cervids with a variety of behavioural and physical changes leading to rapidly advancing morbidity and mortality.

CWD is unusual in that it is found in free ranging animals in their natural environment as opposed to being restricted to livestock [4–10]. Further, as the infectious agent is found not only in neuronal but also in extraneural tissue as well as in bodily fluids and excreta, horizontal transmission is common [5,11]. Transmissibility is exacerbated by the stability of the infectious prions in the natural environment. There are as of today no reported definitive cases of CWD being transmitted to humans [5]. Experiments in humanized mouse models of prion disease have not demonstrated transmission; however, *in vitro* experiments have shown contradictory results and the possibility of future adaption for human transmission exists [5]. Vertical transmission of CWD also occurs in cervids, contributing to disease prevalence [6]. Experimental transmission of CWD to a number of non-cervid (non-humanized) species has been observed, including sheep, rodents, primates, and various carnivores, with canids appearing to be resistant [8]. Vaccination against CWD has been explored as a preventive option, and is still under investigation [12,13] to address this disorder, but success has not yet been achieved. At best, a delay in symptomology in some animals has been observed.

There are several novel approaches for the prevention and treatment of prion disease in humans currently under development. For example, a monoclonal antibody (PRN100) to cellular prion protein has been proposed as a treatment for human prion disease [14]. While not cytotoxic in initial testing, and reaching target drug concentrations, PRN100 treatment still resulted in all patients exhibiting progressive decline in neurological functioning [14]. Thus, while this approach is promising, it is prudent to continue the development of other novel therapies, such as molecular gene therapy approaches.

A potential problem with some proposed pharmacological and immunological therapeutic approaches, in both humans and in animals such as cervids, is the possibility of prion resistance to therapy based on conformational mutation and selection; this has been shown to occur in response to pharmacological therapeutics [15–19]. However, based on experience with Kuru in the Fore people and data from mouse models [20], molecular-directed therapies may be more stable with respect to resistance than are pharmacological or immunological interventions.

These molecular approaches include knockdown of endogenous prion expression [21,22] or overexpression of dominant negative prion forms [20,23,24]. The knockdown option involves depleting levels of the endogenous PrP substrate to reduce PrP availability for conversion, and thus delay disease progression, and has been suggested as a resistance-proof therapeutic strategy [17]. The overexpression option involves utilizing dominant negative exogenous PrP variants to inhibit endogenous PrP conversion and thus suppress disease development. Dominant negative prion variants adopt conformations that inhibit conversion and propagation of pathogenic prion forms [20–25]. The validity of the dominant negative approach is suggested by the existence of naturally occurring PrP variants, presumably existing because of selective pressure, which confer varying degrees of resistance to prion disease [20,25]. Combining overexpression with deletion molecular strategies [24] might be the optimal strategy for both prevention of prion conversion and prevention of resistance to therapy. In addition, resistance can apparently be reversed when the selective pressure is removed, suggesting intermittent changes in therapeutics could be helpful as a strategy against resistance in those cases in which resistance may occur [18]. In the case of molecular therapy, this suggests switching between endogenous PrP depletion and dominant negative PrP expression as a reasonable anti-resistance strategy.

With respect to designing the most appropriate dominant negative PrP molecular approaches for prion disease therapy, it is known that genetic variation in the *PRNP* gene can influence susceptibility to prion disease in humans [20,25], while codon 171 variants in sheep and codon 163 variants in Canidae confer prion disease resistance in those mammals [26]. *PNRP* genetic variation in cervids also influences susceptibility to CWD, but complete resistance has not been observed [6]. It is uncertain if complete resistance could be achieved with overexpression [20,23,24] of any known cervid prion variants; thus, it is possible that native PrP variants would not be suitable for overexpression therapy in cervids. If so, other types of dominant negative PrP variants would need to be used, either those adapted from other mammals [26] or via artificially generated variants, perhaps identified by computational modelling.

With respect to gene therapy for the central nervous system (CNS), an adeno-associated virus (AAV) capsid that binds the human transferrin receptor, which is expressed on the blood‒brain barrier, allows for increased CNS-specific expression in mouse models [27]. Thus, this system is a possible approach for CNS-targeted therapy for prion disease. Therefore, using gene therapy vectors for CNS-directed overexpression is theoretically possible [23,24] and the knockdown methodology is also feasible, with recent *in vivo* data supporting that approach [21,22].

## PROPOSED APPROACH AND TESTING

This paper proposes that CWD should be utilized as a testing target for novel prion disease therapies, particularly those involving knockdown of endogenous prion expression or overexpression of dominant negative prion forms. This would allow testing of these approaches in a natural environment outside of controlled laboratory settings, and if successful would provide direction for human clinical trials; it would alleviate animal suffering in wild cervid populations afflicted with this devastating disease; and would lessen the probability of cross-species transmission to other mammals, including the possibility of adaption for human transmission.

**Figure 1.**
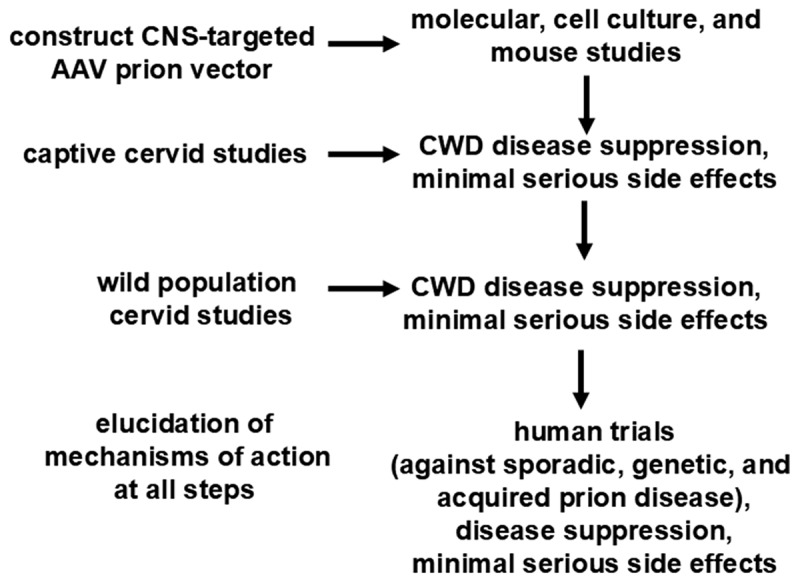
Process of assaying prospective therapies in deer. *In vitro* preliminary work at the molecular and cellular level and *in vivo* work with mice will be followed by *in vivo* experiments on captive cervids in controlled environments, followed by *in vivo* experiments on wild, free-ranging cervids in their natural environment. Optimal endpoints would be disease suppression and minimal serious side effects. Mechanism of action would be determined at each step, if possible. If this work is successful, it would provide further support for human clinical trials.

The general approach would be as follows (Figure 1). Preliminary work would include designing the appropriate vectors and payloads for therapy, such as the therapy approaches discussed in the Introduction. A fundamental initial step would be to identify a PrP variant that would prevent prion conversion and disease progression in cervids. *In vitro* molecular and cell culture experiments could be performed to ascertain if abnormal prion seeding and spread is inhibited by the knockdown or overexpression approaches. However, there is a question whether *in vitro* studies can capture mechanisms of prion resistance using the dominant negative approach; it is possible *in vivo* mouse studies would be required [20,24]. The latter would require a mouse expressing cervid PrP; thus, akin to humanized mouse models, it may be necessary to create a ‘cervidized’ mouse that can serve as an appropriate *in vivo* experimental model for CWD. Since mouse experiments with the human V127 PrP variant suggests dosage effects in which optimized protection is observed with increasing expression of the variant vs. wild-type allele [20], it is possible that a successful gene therapy approach against prion disease would require a combination of methods [24]. First, knockdown of endogenous *PNRP* expression would reduce levels of the vulnerable wild-type PrP, and subsequent overexpression of the dominant negative form would tilt the balance towards optimized disease resistance.

The first *in vivo* experimental step involving the actual target species would be to ascertain if cervids – and we can propose that deer be the specific cervid used – treated with the relevant therapies are resistant to disease under controlled environmental conditions. Captive deer can be treated at four broad intervals: (1) before infectious exposure, (2) at different times after exposure but before any molecular signs of disease, (3) after molecular signs of disease but before symptomology, and then (4) after the first signs of symptomology. These deer would be compared to mock treated controls. Deer in both the experimental and control groups would be stratified as per their *PRNP* gene genetic background. Favourable results from these captive deer experiments can lead to the next steps involving free-ranging wild animals.

Progression from captive deer to wild population deer experiments will proceed only if the captive experiments do not result in significant long-term morbidity for the animals via serious side effects of treatment. Wild deer from areas with significant CWD prevalence can be captured, determined (to the extent possible) to be uninfected with CWD, assayed for genotype, labelled for subsequent tracking, treated with the appropriate therapies, and released back into the wild. Mock treated controls can be processed similarly. Rates of CWD incidence and prevalence in experimental vs. control animals would then be ascertained over time (stratified by genotypic data where available). Given that the incidence of CWD in the wild appears higher in male cervids, likely for behavioural reasons [8], the variable of sex would need to be evaluated in these studies. Neuropathology, immunohistochemistry, and other assays could be performed on post-treatment captured deer to track signs of progression in addition to evaluation of clinical symptomology. At all phases of experimental testing, potential side effects will be monitored. The hoped for outcome is decreased CWD in experimentally treated deer, with the optimal result being full, or at least a large degree of, CWD resistance. In addition, side effects of treatment should be short-term and relatively minimal.

Follow-up studies can attempt to ascertain if the predicted molecular mechanisms are responsible for any observed disease suppression. Success with this work would be further encouragement for the validity of the approach for human clinical trials, building upon experimental laboratory *in vivo* work studies utilizing established mouse models of prion disease.

## CLARIFICATIONS AND OBJECTIONS

Before concluding this manuscript, clarifications of the concept as well as responses to potential objections are presented.

The current manuscript does not propose to use CWD in cervids to *develop* prion disease treatments for humans. The relevant mouse models adequately perform that role. Instead, I propose to utilize CWD in cervids as a testing model to evaluate genetic approaches developed in mice. Further, the current paper does not state that the cervid model is better than other alternative models and so should be used to the *exclusion* of other models. Instead, the argument is that free-ranging wild cervids and CWD represent a testing platform with useful features for evaluating potential human therapies, so that the cervid model is an important *additional* model to use in conjunction with others.

As stated above, the specific utility of the CWD-cervid model is the ability to evaluate novel prion therapies in a free-ranging mammalian population in its natural habitat, independent of a carefully controlled laboratory environment. An objection could be that the environment of wild cervids is quite different from that of humans. This is true, but the same can be said of any other animal model. However, some models are *relatively* more similar to the human condition than others. Thus, free-ranging, genetically diverse cervids interacting with their natural habitat can be considered conceptually more similar to the human condition compared to inbred mouse strains tested in artificial laboratory conditions. Humans are genetically diverse and interact with their environment independent of a controlled experimental protocol. In contrast, domestic cattle and sheep can be considered intermediate between laboratory mice vs. wild cervids. It is true that free-ranging sheep and scrapie can also serve as a natural testing model. However, CWD is an attractive target for testing given the rapidly spreading epidemic in wild cervid populations, particularly in North America, and the urgent need for effective treatments. CWD represents a convergence of practical need with experimental utility.

A related objection would be scepticism as to whether findings from CWD in cervids can be extrapolated to prion disease in humans. Of course, this is an issue with virtually all animal models, including laboratory mouse studies. However, the mechanisms of action for genetic therapies should in theory work against all prion diseases in all species that exhibit such disease. If in fact it is demonstrated that the results do not carry over from one species to others then this is an important data point that can inform directions for subsequent further experiments and optimization. If a general genetic approach works in the laboratory but not in the wild, this can serve as a warning that more work may be needed before human clinical trials.

Another objection to the proposed CWD-focused approach would be to question why we should focus on a prion disease not (currently) transmissible to humans, rather than on animal prion diseases that are. This can be answered three ways. First, nothing in the present manuscript precludes testing on other models of prion disease, including forms known to be transmissible to humans. Thus, the proposed genetic approach should work against vCJD in humans as well as BSE in cattle. As stated, the CWD-cervid testing model is not meant to be exclusive of others, but rather an important addition to other types of analysis. Second, the lack of (current) human transmissibility can be viewed as a safety advantage in conducting these experiments. Third, the potential for future transmissibility is a rationale for dealing with the CWD epidemic before such an eventuality occurs.

There is an urgent need for new human prion therapies. At the same time, there is also an urgent need to address the CWD epidemic. Novel genetic therapies aimed at inhibiting aberrant prion propagation in the CNS can serve both roles. For the reasons stated in this manuscript, testing these therapies in cervids against CWD would be a prudent additional step before human clinical trials. There is no compelling reason not to attempt this and no compelling reason to reject the opportunity to obtain potentially useful information for human trials while at the same time addressing the CWD crisis.

## DISCUSSION

The proposed work has three potential practical benefits. First, using CWD as a natural environment, ‘real life’ model for testing novel preventive and therapeutic approaches against prion disease is of benefit to further evaluate approaches that could have potential human clinical utility. Unlike laboratory mice or livestock, free-ranging wild cervids have a more natural interaction with the environment and thus can more closely mimic the human situation in which prion disease can be acquired outside of carefully controlled conditions.

Thus, success would be an encouraging step towards human clinical trials. Second, from the standpoint of ethical promotion of animal well-being, effective treatment for CWD can alleviate significant suffering in cervid populations, decreasing morbidity and mortality from this disease. Thus, treating CWD in cervids is a positive step in its own right, independent of potential utility for human clinical testing. If molecular therapies work, there is an ethical imperative to alleviate animal suffering. Further, a similar approach can be used for other animal species that suffer from prion disease (e.g., sheep, cattle). As described above, alternative approaches, such as vaccination, have not been sufficiently effective and can in theory be countered by prion therapy resistance. Third, by decreasing the prevalence of prion disease in cervids, the risk of potential transmission to humans and other species [8,11,28] can be lessened.

## Data Availability

No materials were used for this manuscript, and no experiments were performed to produce this manuscript.

## CRediT

This manuscript is wholly the work of MB. MB conceptualized, wrote, and edited the manuscript.

## Code availability

No code has been utilized or described in this article.
